# Responses to Dehydration in the One-Humped Camel and Effects of Blocking the Renin-Angiotensin System

**DOI:** 10.1371/journal.pone.0037299

**Published:** 2012-05-18

**Authors:** Mahmoud Alhaj Ali, Abdu Adem, Irwin S. Chandranath, Sheela Benedict, Javed Y. Pathan, Nicolas Nagelkerke, Fred Nyberg, Lynley K. Lewis, Tim G. Yandle, Gary M. Nicholls, Chris M. Frampton, Elsadig Kazzam

**Affiliations:** 1 Department of Pharmacology, Faculty of Medicine and Health Sciences, United Arab Emirates University, Al Ain, United Arab Emirates; 2 Department of Pharmaceutical Biosciences, Uppsala University, Uppsala, Sweden; 3 Department of Internal Medicine, United Arab Emirates University, Al Ain, United Arab Emirates; 4 Department of Community Medicine, United Arab Emirates University, Al Ain, United Arab Emirates; 5 Department of Medicine, Christchurch Hospital, Otago University, Christchurch, New Zealand; Universidad Europea de Madrid, Spain

## Abstract

Our objectives were to compare the levels of circulating electrolytes, hormones, and renal function during 20 days of dehydration in camels versus the level in non-dehydrated camels and to record the effect of blocking angiotensin II AT1 receptors with losartan during dehydration. Dehydration induced significant increments in serum sodium, creatinine, urea, a substantial fall in body weight, and a doubling in plasma arginine vasopressin (AVP) levels. Plasma aldosterone, however, was unaltered compared with time-matched controls. Losartan significantly enhanced the effect of dehydration to reduce body weight and increase serum levels of creatinine and urea, whilst also impairing the rise in plasma AVP and reducing aldosterone levels. We conclude that dehydration in the camel induces substantial increments in serum sodium, creatinine, urea and AVP levels; that aldosterone levels are altered little by dehydration; that blockade of angiotensin II type 1 receptors enhances the dehydration-induced fall in body weight and increase in serum creatinine and urea levels whilst reducing aldosterone and attenuating the rise in plasma AVP.

## Introduction

The one-humped camel (*Camelus dromedarus*) is widely distributed in the Gulf countries. It is well known that camels are able to survive water deprivation for long periods without ill effects [1]. The camel's physiological adaptation to the desert environment is due to the extremely low rate of water turnover, which is accomplished by minimal use of evaporative cooling, low urinary output, and its ability to extract water from undigested feed residues [2], [3]. Furthermore, camels can change their body temperature according to changes in the ambient temperature as a water saving mechanism [4]. It has been found that the camel can tolerate a loss of water corresponding to 30% of its body weight when dehydrated whereas other mammals may die from circulatory failure when the water loss involves 12% of their body weight [1], [4].

Drinking is controlled by a number of factors including tissue osmolality and more importantly blood osmolality [5]. The circulating and central renin-angiotensin systems (RAS) also contribute to water and sodium balance through a constellation of actions and interactions. Angiotensin II (Ang II) acts through two receptor subtypes, AT1 and AT2, that have been cloned and characterized pharmacologically [6], [7]. Most of the classic physiological effects of Ang II, such as sodium and water retention, vasoconstriction, and aldosterone and vasopressin release are mediated by the AT1 receptor [8].

The maintenance of fluid, electrolyte and circulatory homeostasis during dehydration is likely to be partly dependent, upon changes in circulating levels of hormones with known effects on sodium and water balance. Whereas the effect of dehydration on some of these hormones are well documented in certain species, but not so for the camel. Accordingly, we studied the effects of dehydration in camel plasma levels of sodium and water-retaining hormone systems including the renin-angiotensin system, aldosterone and antidiuretic hormone. We investigated the role of the renin-angiotensin system in plasma electrolytes, renal function and hormonal responses after long term (20 days) dehydration with or without AT1 receptor blocker (losartan) in a subset of dehydrated camels. Our hypothesis was that the renin-angiotensin system contributes to the maintenance of renal function, sodium, potassium and water homeostasis, aldosterone and antidiuretic hormone secretion during dehydration.

## Materials and Methods

The study protocol was approved by the Animal Ethics Committee of the United Arab Emirates University. The approval ID was AE/03/38. The camels were visited daily by an experienced Veterinarian to ensure their well being. Thirty male camels, aged 3–4 years and weighing 290–348 kg were studied. They were kept under shade in a corral during the summer months of June and July when temperatures varied between 40 and 48°C. They were divided into three groups: control camels, 6 in number, allowed free access to feed and water during the 20-day study: 18 camels were denied water access but given food *ad libitum* for 20 days and 6 camels were similarly dehydrated but in addition were given losartan (Merck, NJ, USA), 5 mg/Kg body weight daily by injection into an external jugular vein. All camels were maintained on dry hay for the first week of the experiment and green-hay for the remainder of the study. Body weights were calculated in all animals at baseline and again on days 5, 10, 15 and 20 using the formula: weight (Kg)  =  shoulder height × chest girth × hump girth ×50 [9]. The three groups of animals were studied simultaneously. Blood samples for the measurement of serum electrolytes, urea and creatinine were collected from the external jugular vein between 08:00–10: 00 hours, two days before the start of the experiment (baseline) and again on days 5, 10, 15 and 20 days of dehydration. Blood was collected in two vacutainers with and without anticoagulant. Analyses were carried out using an ACE chemistry analyzer (Alfa Wassermann, NJ, USA) within one hour of sampling. Venous samples for hormone measurements were drawn simultaneously at baseline and days 5, 10 and 20 of dehydration or control. The blood was taken into vacutainers on ice containing K_3_-EDTA, centrifuged at +4°C within one hour and the plasma stored as aliquots at−80°C until analyzed. Assays for plasma hormones were performed using commercial radioimmunoassay kits as follows: aldosterone and arginine vasopressin (AVP) all from Diagnostics Systems Laboratories Inc., TX, USA, and plasma renin activity (PRA) from DiaSorin, MN, USA. Hormone levels from any one camel were measured in the same assay to avoid inter-assay variability. The intra-assay coefficients of variation varied between 4.5% for aldosterone and 7% for cortisol. Cortisol is reported with other stress hormones in a separate study. In order to determine whether the dose of losartan administered was sufficient to affect the binding of angiotensin II to AT1 receptors, liver tissue was obtained upon completion of the study, at slaughter, from 3 camels in each of the three study groups. Measurement of angiotensin II receptor binding to camel liver and intestine was determined using an angiotensin II binding assay [10]. The specific binding of ^125^I-Angiotensin II (0.36 nM) was displaced by different concentrations of unlabelled. Angiotensin II (0.001–100 nM) in the presence of 1 uM losartan (AT1 receptor selective antagonist) or 1 uM PD-123319 (AT2 receptor selective antagonist). Ki values were calculated from IC50 values using the Cheng and Prusoff equation:

Ki  =  IC50/1+ [L]/Kd.

### Statistical Analysis

For assessment of angiotensin II binding to hepatic tissue, the mean±SEM is given from three experiments each performed in triplicate. Differences between the 3 groups of camels for body weight, serum biochemistry and plasma hormone levels were determined using 2-way analysis of variance (ANOVA) with time as a repeated measure factor. Where significant differences were identified by ANOVA, the level of significance at individual time points was established by Fisher's protected least-significant difference tests, using the appropriate mean-square error term from the ANOVA. Data are shown as mean±SEM, and statistical significance was assumed at p<0.05.

## Results

### Effects of dehydration and losartan

Angiotensin II receptors in the camel liver were exclusively of the AT1 subtype. The displacement studies showed a marked 16-fold increase (p<0.0001) and 14-fold decrease (p<0.0001) in the dehydrated (Ki  = 0.06±0.005 nM) and losartan-treated (Ki  = 13.3±1.0 nM) camels, respectively compared to the controls (Ki  = 0.98±0.001 nM). Calculated body weight increased slightly over the 20 day study period in control animals (from 302±14 to 318±12 Kg), decreased by 34.7% across 20 days in dehydrated camels (from 291±5 to 190±2 Kg) and by 38.8% (from 348±15 to 213±5 Kg) in losartan-treated/dehydrated camels.

Changes in body weight between control and the two dehydrated groups were statistically significant (P<0.0001), and the change in losartan-dehydrated animals was significantly greater than in the dehydrated-only group (P<0.0001). Baseline levels of serum sodium were similar in control, dehydrated and losartan-treated camels (141.3±1.2, 144.2±1.2 and 144.6±0.7 mmol/L, respectively). Whereas serum sodium levels did not change significantly at day 20 in control animals, they increased significantly (p<0.001) in both losartan-treated and dehydrated camels (Table 1). Although the rise in serum sodium overall was greater in losartan-treated animals versus dehydrated camels, the differences were not statistically significant between the two dehydrated groups. Serum potassium concentrations were similar in the three groups at baseline (4.0±0.2, 4.5±0.1, and 4.3±0.2 mmol/L for control, losartan-treated and dehydrated camels, respectively) and were unchanged at day 20 in all camels.

**Table 1:** Plasma levels of Na, Urea and Creatinine in non-dehydrated (“control”, n = 6), Losartan treated (n = 6), and dehydrated camels (n = 18) on Basal day and on day 20 of dehydration.

	Group of camels	Base-line level	Day 20
**Na**	Control	141.3±1.2	145.2±1.02
**(mM/L)**	Losartan	144.2±1.2	180.7±4.3***
	Dehydrated	144.6±0.7	175.3±2.2***
**UREA**	Control	15.7±1.3	11.2±1.3
**(mg/dL)**	Losartan	16.4±0.8	39.2±6.8**
	Dehydrated	13.3±1.4	34.9±3.1**
**CREATININE**	Control	1.43±0.11	1.5±0.1
**(mg/dL)**	Losartan	1.33±0.07	2.5±0.37*
	Dehydrated	1.28±0.13	2.2±0.1*

Data are shown as Mean±SEM. Significant difference from control is denoted by *p<0.05, **p<0.01 and ***p<0.01.

**Figure 1:**
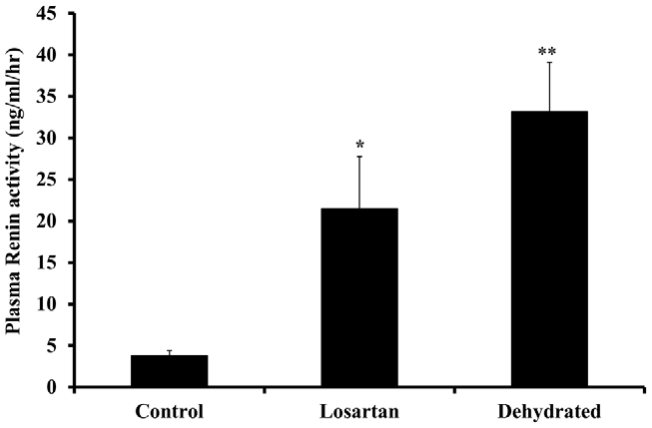
Plasma renin activity in non-dehydrated (“control”, n = 6), Losartan treated (n = 6), and dehydrated camels (n = 18) on day 20 of dehydration. Data are shown as mean ±SEM. Significant difference from control is denoted by *p<0.05, **p<0.01.

Serum urea levels were not different between control, losartan-treated and dehydrated animals at baseline (15.7±1.3, 16.4±0.8 and 13.3±1.4 mg/dL, respectively) and did not change significantly at day 20 in the control group. Compared with control animals, serum urea levels increased significantly (p<0.001) in both dehydrated groups at day 20 of dehydration (Table 1).

Serum creatinine concentrations likewise were similar in the three study groups at baseline (1.43±0.11, 1.33±0.07 and 1.28±0.13 mg/dL in the control, losartan-treated and dehydrated camels, respectively) and were unaltered by day 20 in the control group. As with urea, serum creatinine levels increased significantly (p<0.001) over 20 days in both groups of dehydrated camels compared with time-matched controls (Table 1). Furthermore, the change in creatinine from baseline was significantly greater in both dehydrated camels with and without losartan.

**Figure 2:**
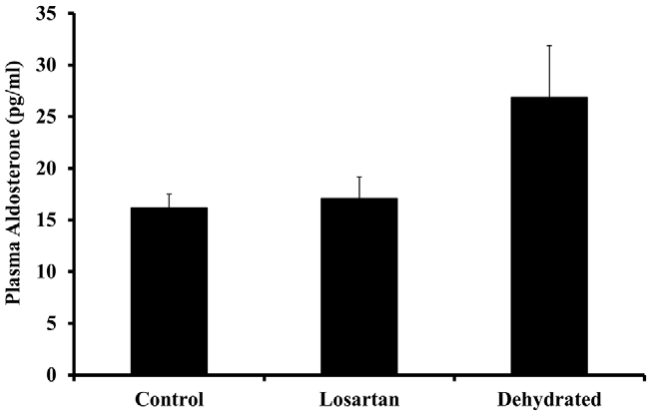
Plasma aldosterone in non-dehydrated (“control”, n = 6), Losartan treated (n = 6), and dehydrated camels (n = 18) on day 20 of dehydration. Data are shown as mean±SEM. Changes in aldosterone level in dehydrated camels is not statistically significant compared to control camels.

**Figure 3:**
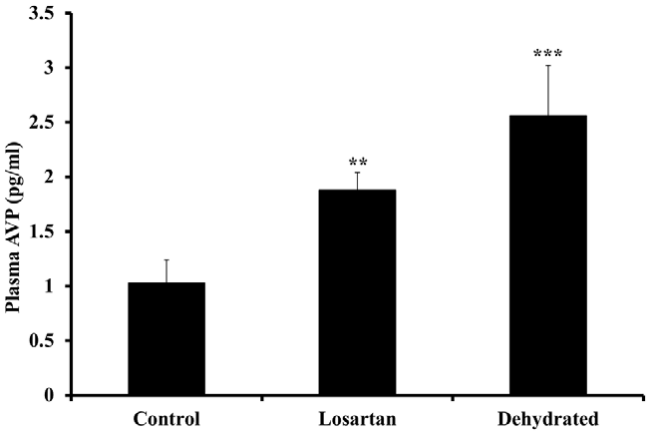
Plasma arginine-vasopressin in non-dehydrated (“control”, n = 6), Losartan treated (n = 6), and dehydrated camels (n = 18) on day 20 of dehydration. Data are shown as mean±SEM. Significant difference from control is denoted by **p<0.01 and ***p<0.01.

Compared with the control group, the levels of plasma renin activity increased significantly at day 20 of dehydration with losartan (p<0.05) and for the dehydrated without losartan (p<0.01) with no significant difference between the two dehydrated groups (Fig. 1). Whereas plasma aldosterone concentrations remained steady over 20 days in control animals they increased variably but not statistically significant in both dehydrated groups (Fig. 2). The modest rise in aldosterone levels in the dehydrated camels contrasted with a decline in losartan- treated camels. Compared with control camels, plasma arginine vasopressin concentrations rose significantly in both losartan-treated and dehydrated camels (Fig. 3).

## Discussion

The camel has an extraordinary capacity to survive water deprivation. This is likely to be explained, at least in part, by its ability to alter the production of hormones which are capable of altering sodium and water homeostasis. The hormone system including AVP, aldosterone and the renin-angiotensin system, which under most circumstances, serve to retain water and/or sodium and maintain circulatory and renal status. Although earlier studies in camels have documented responses in AVP, renin-angiotensin system and aldosterone to dehydration [11], , the duration of these studies has been relatively brief (8–14 days), time-matched controls (non-dehydrated animals) were not used. In this study, we have documented changes in all of these hormones after a longer period (20 days) of water deprivation with time matched measurements in non-dehydrated camels. We hypothesized that the renin-angiotensin system might play a central role in maintaining homeostasis during dehydration. If this hypothesis is correct we should expect to see greater disturbances in water balance, electrolyte levels and renal function in dehydrated camels when the renin-angiotensin system is blocked with losartan. A liver receptor study was carried out in order to determine whether the dose of losartan chosen was sufficient to block, at least in part, angiotensin II type AT1 receptors. The results indicate that indeed there was an effect of losartan on these receptors, at least in the liver. As anticipated, dehydration induced a substantial decline in body weight and an increase in serum sodium to levels that would probably prove fatal in most other animals. Likewise, and as reported previously, dehydration resulted in an increase in serum urea and creatinine reflecting, presumably, a major decline in glomerular filtration rate [13]. That the renin-angiotensin system is important to the maintenance of water balance during dehydration is suggested by the significantly greater fall in body weight and rise in serum creatinine and urea levels (and a trend for higher serum sodium levels) with the administration of losartan.

In congruence with earlier observations [14], [12], we noted little change in circulating levels of aldosterone in dehydrated camels despite vigorous activation of the renin-angiotensin system. One possible explanation for this minimal response in circulating aldosterone levels in dehydrated animals is the substantial rise in serum sodium and presumed, though not measured, increase in plasma osmolality. Studies from the 1960s and 1970s in experimental animals and man demonstrated that an increase in extracellular sodium concentration can itself suppress aldosterone production and also inhibit the stimulatory actions of its known secretagogues. For example, an increase in the plasma sodium concentration of 6–15 mmol/L in blood supplying the auto-transplanted sheep adrenal gland inhibited the aldosterone secretory response to sodium depletion [15]. Furthermore, the aldosterone secretory response to infused angiotensin II in this sheep model was inversely related to the plasma sodium concentration [16]. Increases in plasma sodium concentration of lesser magnitude than in the current study were shown to inhibit the aldosterone secretory response to angiotensin II or potassium by the isolated perfused canine adrenal gland [17]. Furthermore, studies in healthy humans [18] and in a patient with diabetes insipidus and adipsia [19] indicate that an increase in serum sodium concentration reduces aldosterone levels and inhibits it responsiveness to known stimuli. Finally, support for a modulating effect of circulating sodium concentration on aldosterone secretion comes indirectly from studies of volume depletion in camels induced by the intravenous injection of furosemide where, in contrast to the present situation of water depletion, plasma sodium levels declined [20] and PRA and plasma aldosterone levels increased vigorously (more than 4-fold) and in parallel.

In the present study, dehydration stimulated a significant increase in plasma AVP. The most obvious explanation for this is the rise in serum sodium (and osmolality) together with the inevitable, though unmeasured fall in circulating volume and arterial pressure. It is possible also that the substantial increase in activity of the renin-angiotensin system contributed since high concentrations of angiotensin II are capable of stimulating AVP secretion [21], an effect enhanced during water deprivation and inhibited by water-loading [21], [22]. Some support for this contention comes from our observation that plasma AVP levels were lower after 20 days of dehydration in camels receiving losartan compared with those without losartan. Albeit, no significant differences were seen between the two dehydrated groups.

Some limitations of our study deserve mention. We would have preferred direct measurements of body weight over derived values based on a number of length measurements each with their own potential for error. We cannot be sure from our study whether the blockade of angiotensin II Type-1 receptors with the dose of losartan we chose was complete or incomplete. We have presumed that measurements of PRA reflect accurately the degree of activation of the renin-angiotensin system whereas ideally, we should like to have measured also levels of angiotensin II, the major end-product of the system. Another limitation in our study was assessment of the degree of angiotensin II type 1 receptor blockade using liver, rather than adrenal tissue. If blockade of adrenal receptors was minimal with the dose of losartan used, we may have underestimated the role of the renin-angiotensin system in regulating aldosterone secretion under the conditions of our study. Finally, overcoming the challenge of measuring urinary volume and electrolytes in dehydrated camels would have provided useful data with which to relate to circulating hormone levels.

In conclusion, 20 days of dehydration induced major increments in serum sodium, urea and creatinine, a marked decline in body weight along with activation of the renin-angiotensin system, and increased circulating levels of AVP. Plasma levels of aldosterone were slightly altered despite stimulation from its known secretagogues presumably the result of the rise in serum sodium level. Since responses to dehydration in body weight, serum creatinine and urea, plasma aldosterone and AVP were altered by losartan administration, it is probable that the renin-angiotensin system contributes to the maintenance of circulatory homeostasis and kidney function during dehydration in the camel.
